# Severe immunotherapy-related thrombocytopenia in metastatic bone cancer: a multicenter retrospective case series highlighting early recognition and management

**DOI:** 10.3389/fonc.2025.1574379

**Published:** 2025-05-27

**Authors:** Qian Gao, Zhaonong Yao, Yuhong Yao, Yunxia Liu, Jianshui Mao, Binghao Li

**Affiliations:** ^1^ Nursing Department, The Second Affiliated Hospital of Zhejiang University School of Medicine, Hangzhou, China; ^2^ Department of Orthopaedics, The Second Affiliated Hospital of Zhejiang University School of Medicine, Hangzhou, China; ^3^ Department of Medical Oncology, Hangzhou Third People’s Hospital, Hangzhou, China; ^4^ Department of Orthopaedics, The Fourth Affiliated Hospital of Zhejiang University School of Medicine, Yiwu, China

**Keywords:** immune checkpoint inhibitor, immunotherapy, thrombocytopenia, precision nursing care, PD-1

## Abstract

**Background:**

Thrombocytopenia (TP) is a rare adverse event (<1%) associated with immune checkpoint inhibitor (ICI) therapy, termed immunotherapy-related thrombocytopenia (irTP). This condition is particularly concerning in patients with metastatic bone cancer due to the increased risk of life-threatening bleeding complications, which may further compromise patient management. Moreover, the scarcity of systematic reports on irTP in this population underscores the need for focused investigation.

**Methods:**

We retrospectively reviewed the clinical records of patients with metastatic bone cancer who received single-agent ICI therapy—specifically, PD-1 inhibitors such as pembrolizumab, penpulimab, and sintilimab—between May 2020 and December 2024 at three tertiary hospitals. Patients who developed severe irTP were included in the analysis.

**Results:**

A total of 94 cases were screened, of which 7 patients (7.45%) were diagnosed with severe irTP. The primary cancer subtypes included melanoma (n = 4), undifferentiated pleomorphic sarcoma (n = 2), and renal clear cell carcinoma (n = 1). All seven patients were undergoing combination therapy with the bone anti-resorptive agent denosumab. The median time to irTP onset was 92 days after the initial ICI administration. Following diagnosis, all patients were hospitalized and received intensive immunomodulatory therapy, supportive care, and meticulous nursing management. While symptoms significantly improved in all cases, long-term follow-up indicated that patients remained stable after discharge. The median duration of hospitalization was 18 days.

**Conclusions:**

Although irTP is considered rare in the literature, we observed an incidence of 7.45% in our cohort, highlighting a significant clinical concern due to the potential for severe bleeding complications in metastatic bone cancer patients. Timely diagnosis and comprehensive treatment—supported by interdisciplinary collaboration among oncologists, hematologists, and nursing staff—are essential for achieving full recovery. Furthermore, our findings emphasize the need for future research into predictive biomarkers and risk factors for irTP.

## 1 Introduction

Immune checkpoint inhibitor therapy, particularly with programmed cell death-1 (PD-1) antibodies, has emerged as a cornerstone in the management of advanced solid tumors (1, 2). However, this therapeutic breakthrough comes at the cost of unique an immune-related adverse events (irAEs) that differ from the toxicities associated with traditional chemotherapy or targeted therapies (3–6). Although the most commonly reported irAEs include dermatologic reactions, fatigue, and gastrointestinal disturbances, hematologic toxicities—while less frequent—can be particularly severe and even life-threatening (7–9). Among these, immunotherapy-related thrombocytopenia (irTP) is particularly concerning due to the potential for spontaneous bleeding, increased risk of severe hemorrhage, and complications related to bone marrow suppression.

Recent case reports have underscored that PD-1 antibodies may lead to severe bone marrow suppression, resulting in profound irTP and, in some instances, fatal outcomes (10, 11). The early manifestations of hematologic toxicity can be subtle and may overlap with side effects induced by other medications, potentially leading to delays in recognition and intervention by clinical staff (12, 13). However, the mechanisms underlying irTP remain incompletely understood. In the context of bone metastases, it is hypothesized that the interplay between PD-1 blockade, the tumor microenvironment, and bone-targeted therapies such as denosumab may further exacerbate platelet depletion.

Given the clinical importance of early recognition and intervention, both physicians and nursing staff play pivotal roles in the observation, identification, and targeted management of these AEs (14). While comprehensive care strategies for irAEs have been reported (9), data on severe irTP in patients with bone metastases remain scarce. This gap in knowledge is clinically significant, as these patients are already at increased risk for skeletal complications, and the added burden of thrombocytopenia may complicate their overall treatment course. The challenges in recognizing and managing irTP highlight the need for timely identification, multidisciplinary intervention, and evidence-based treatment strategies.

This study retrospectively analyzed the treatment, management, and nursing care processes for patients who developed irTP after PD-1 antibody immunotherapy between May 2020 and December 2024 at three tertiary hospitals in Zhejiang Province. By characterizing the treatment responses and outcomes, we aim to improve understanding of irTP in this population and provide evidence-based guidance for optimizing immunotherapy safety and efficacy.

## 2 Materials and methods

### 2.1 Patient selection and data collection

A retrospective review was conducted involving patients with cancer bone metastasis who were treated with a single-agent PD-1 antibody between May 2020 and December 2024 at three tertiary hospitals in Zhejiang Province, China. No formal hypothesis testing was conducted given the case series nature of the study. Patient data were retrospectively extracted from medical records and included demographics (age, sex), treatment history, and performance status. Disease-specific characteristics, such as histological subtype, PD-L1 expression status, lesion localization, number, and dimensions of bone metastases, were also recorded. In addition, details regarding the management of bone metastases—including whether patients underwent surgical intervention or radiotherapy—and the administration of supportive therapies were documented.

Patients receiving combination anticancer therapies, such as chemotherapy, were excluded from the study. Similarly, individuals with a preexisting history of thrombocytopenia, bleeding disorders, other systemic hematologic disorders or autoimmune diseases were also excluded from analysis to avoid confounding variables. Post-discharge, patients were followed up every three months for a minimum of 12 months or until the last available contact, with scheduled evaluations that included clinical examinations, laboratory tests (including platelet counts), and imaging as indicated. Data collected during follow-up included the total duration of hospitalization, the time interval between the first immune checkpoint inhibitor (ICI) treatment and subsequent hospital admissions, overall survival, and any long-term complications or recurrences of TP.

### 2.2 Therapeutic procedures and AE assessment

All therapeutic procedures were performed by certified physicians with extensive experience in oncology and immunotherapy. Adverse events were graded according to the Common Terminology Criteria for Adverse Events (CTCAE) version 4.0. Severe adverse events (SAEs) were defined as any adverse event of grade ≥3 or any irAE known to be associated with ICI therapy. Management of irTP was conducted in accordance with the Chinese Society of Clinical Oncology (CSCO) Guidelines for Toxicity Management Related to Immune Checkpoint Inhibitors. Briefly, immunomodulatory therapy included high-dose corticosteroids as the first-line treatment. Subcutaneous recombinant thrombopoietin (TPO) was administered to stimulate platelet production. Patients with grade 3 toxicity were initiated on methylprednisolone at 1 mg/kg/day, while those with grade 4 toxicity received a higher dose of 2 mg/kg/day. For patients with grade 4 toxicity, additional intravenous immunoglobulin (IVIG) was also administered at a dose of 20 g/day. Escalation to mycophenolate mofetil was considered if clinical deterioration was observed after corticosteroid and IVIG adimistration after one week. In situations where platelet counts fell below 5 × 10^9/L, platelet transfusions (10 U) were provided as a supportive measure.

### 2.3 Statistical analysis

Descriptive statistics were used to summarize the data. All analyses and data illustration were performed using Graphpad Prism 9.0. Statistical significance testing was not conducted because no comparison was performed.

## 3 Results

### 3.1 Study population

During the study period from May 2020 to December 2024, a total of 94 patients with bone metastasis who received single-agent PD-1 antibody therapy were screened. Of these, 8 patients (8.51%) developed irTP of all grades. Among them, 7 (7.45%) developed severe thrombocytopenia (TP) and were subsequently included in further analysis. The cohort consisted of 3 male and 4 female patients, with ages ranging from 44 to 72 years. Baseline laboratory evaluations, including complete blood counts, cardiopulmonary assessments, and liver and kidney function tests, were within normal limits for all patients. None of the individuals had a documented history of autoimmune disorders or contraindications to PD-1 antibody therapy.

The primary malignancies included melanoma (n = 4), undifferentiated pleomorphic sarcoma (n = 2), and renal clear cell carcinoma (n = 1). In terms of symptom grading, one patient exhibited grade 3 thrombocytopenia throughout the clinical course, one patient initially presented with grade 3 toxicity that escalated to grade 4 during hospitalization, and the remaining five patients were classified as grade 4 at the time of diagnosis. The onset of severe thrombocytopenia ranged from 32 to 144 days after the first infusion of a PD-1 antibody. The PD-1 inhibitors administered included pembrolizumab (n = 2), penpulimab (n = 3), and sintilimab (n = 2). Additionally, four patients underwent concurrent radiotherapy for bone metastases, and all patients received monthly subcutaneous injections of denosumab (120 mg) as part of bone protective therapy. Patient demographics was illustrated in **Table 1**.

**Table 1:** Patient demographics.

Case number	Age	Sex	Pathology	Antibody	Onset	Grade	PD-L1*	Radio	Surgery	Prior medication	ECOG	Comorbidities
Case 1	57	Male	Melanoma	Pembrolizumab	92 days	3	Negative	Yes	Yes	Dabrafenib with trametinib	1	Hypertension
Case 2	44	Female	Melanoma	Penpulimab	33 days	4	10%	No	Yes	Temozolomide	0	N/A
Case 3	59	Female	UPS	Sintilimab	104 days	4	25%	Yes	Yes	Doxorubicin	1	Diabetes
Case 4	72	Female	UPS	Penpulimab	47 days	4	Negative	Yes	No	N/A	0	Hypertension
Case 5	47	Male	Melanoma	Pembrolizumab	60 days	4	Negative	No	No	N/A	1	N/A
Case 6	48	Male	Melanoma	Sintilimab	84 days	3→4	1%	Yes	No	Temozolomide	1	N/A
Case 7	56	Female	RCC	Penpulimab	144 days	4	> 20%	No	Yes	Lenvatinib	1	Diabetes

UPS, undifferentiated pleomorphic sarcoma. RCC, renal clear cell cancer. ECOG, Eastern Cooperative Oncology Group performance status scale.

*CPS method, combined positive score of PD-L1 immunohistochemistry 22C3 staining.

### 3.2 Clinical presentation and treatment interventions

Among the 7 patients, the individual with grade 3 thrombocytopenia did not exhibit any clinical symptoms, although laboratory tests revealed a significant drop in platelet count (initial count: 46 × 10^9/L). In contrast, six patients experienced gingival bleeding during routine activities such as tooth brushing. Four patients reported spontaneous epistaxis, and one patient presented with widespread petechiae and ecchymoses. Importantly, none of the patients exhibited more severe bleeding complications such as hematuria, hematochezia, or neurological symptoms indicative of intracranial hemorrhage. Coagulation studies in six patients revealed markedly prolonged prothrombin time (PT) and activated partial thromboplastin time (APTT), indicating a disturbance in the coagulation cascade.

Upon emergency admission, the patient history, including recent PD-1 antibody therapy, prompted clinicians to manage the patients according to CSCO Guidelines for irAE management. Six patients presented elevation in platelet counts in one week after corticosteroid administration with or without intravenous IVIG. However, one patient (Case 6) with grade 4 toxicity failed to achieve an adequate platelet response; in this instance, the steroid dosage was escalated to 500 mg/day for three days and mycophenolate mofetil (Cellcept) was added to the regimen. The therapeutic decision was made according to the CSCO Guidelines for irAE management. This patient was also closely monitored with contingency plans for potential transfer to the intensive care unit (ICU). Six days after the initiation of mycophenolate mofetil, the platelet counts were elevated to over 50 × 10^9/L.

**Figure 1:**
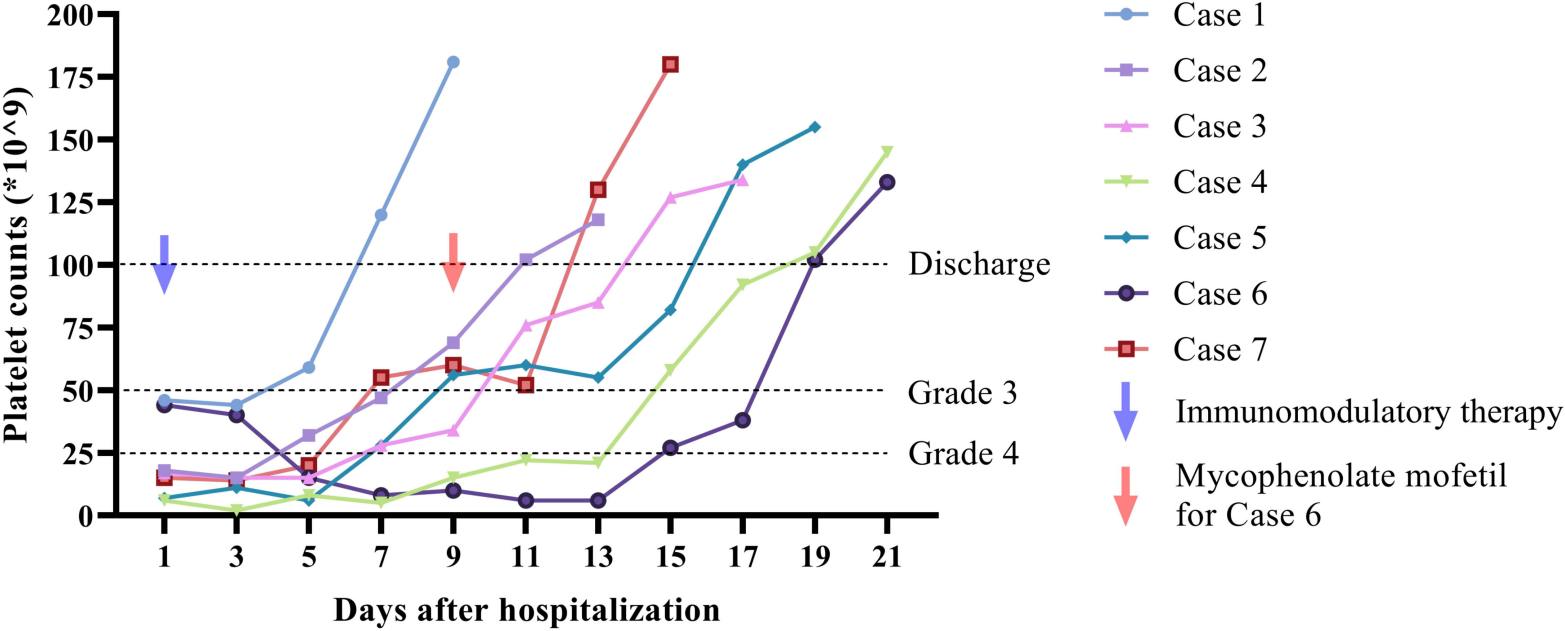
Dynamic change of platelet counts during hospitalization of 7 included cases. Five of the cases showed rapid response to immunomodulatory therapy on day 5 after hospitalization. One patient (case 4) presented elevation of platelet counts on day 9. One patient showed no response after treatment with corticosteroid and IVIG. Addition of mycophenolate mofetil was initiated on day 9 and respond was monitored on day 15. Patients were discharged when the platelet counts were over 100 × 10^9/L. One follow up of platelet counts was presented in the figure.

Supportive care measures, including active fluid replacement and gastric protection, were administered concomitantly during steroid therapy. Upon discharge, all patients demonstrated significant clinical improvement, with platelet counts rising to levels exceeding 50 × 10^9/L and the resolution of associated symptoms such as bleeding, fatigue, and gastrointestinal disturbances. Following discharge, PD-1/PD-L1 antibody therapy was permanently discontinued, and corticosteroid dosages were gradually tapered in accordance with medical guidance. Follow-up evaluations indicated that 7 patients maintained normal platelet counts (100–300 × 10^9/L) during the observation period. The dynamic changes of platelet counts of patients were illustrated in **Figure 1**.

### 3.3 The recognition and comprehensive management of AEs

Routine blood tests revealed a progressive decline in platelet counts and a concomitant reduction in fibrinogen levels. After excluding other potential contributing factors, such as the effects of radiotherapy, PD-1/PD-L1 antibody–induced thrombocytopenia was diagnosed. Upon admission, an integrated irAE management team was immediately assembled. This team comprised orthopedic oncology and tumor internal medicine specialist nurses as well as responsible bedside nurses. A comprehensive patient file was created, which included details of the patient’s general health status, medication history, and the initiation date of immunotherapy. Detailed records were maintained regarding the onset, frequency, severity, and type of thrombocytopenia, as well as associated clinical symptoms such as bleeding.

The interdisciplinary team provided extensive health education to patients and their families, focusing on the prevention and management of irAEs, particularly thrombocytopenia. Prior to initiating treatment, specialist nurses explained precautionary measures and provided educational materials to guide patients in recognizing early signs of bleeding and other adverse events. After discharge, responsible nurses conducted telephone follow-ups on the 7th, 14th, and 28th days to closely monitor the patients’ conditions and ensure continuity of care.

The nursing care strategy incorporated meticulous bleeding prevention measures, including the placement of patients in a low-traffic, quiet environment to reduce physical trauma. During routine procedures such as blood draws and intravenous punctures, nurses employed gentle techniques and minimized tourniquet time, ensuring prolonged compression post-procedure to mitigate bleeding risks. Environmental conditions were optimized by maintaining room temperatures between 18°C and 22°C with a relative humidity of 60%, thus preventing mucosal dryness that might precipitate bleeding. In addition, dietary recommendations were provided to encourage a high-protein, high-vitamin, easily digestible diet while avoiding irritants such as spicy or greasy foods that could exacerbate bleeding risks during defecation.

When platelet counts fell below critical levels (20 × 10^9/L), nurses were instructed to vigilantly monitor for spontaneous bleeding and to assess for early signs of intracranial hemorrhage, such as headache, nausea, blurred vision, or altered consciousness. This proactive approach allowed for the timely identification and management of potentially life-threatening complications.

### 3.4 Nursing management during corticosteroid therapy and beyond

Patients undergoing corticosteroid treatment were also provided with prophylactic medications to mitigate steroid-related side effects (15). These included oral calcium carbonate D3 tablets, calcifediol soft capsules, and sustained-release potassium chloride tablets, as well as intravenous esomeprazole to protect gastrointestinal mucosa. Prior to initiating oral prednisone, nurses collaborated with physicians to assess for any contraindications, including a history of severe psychiatric conditions, gastrointestinal surgeries, uncontrolled hypertension, diabetes, or a predisposition to infections. Dietary modifications were implemented to address electrolyte imbalances, emphasizing a high-potassium, high-protein, and low-sodium (less than 6 g/day) regimen. Regular monitoring of blood pressure, blood glucose, and electrolyte levels was performed, with any abnormalities promptly reported to the attending physician.

The nursing team also placed significant emphasis on providing emotional support to patients. Many of the patients in this series, being advanced cancer patients with bone metastasis, exhibited signs of anxiety, depression, and emotional distress. Nurses engaged in empathetic communication, actively listening to patient concerns and providing reassurance regarding the treatment plan. In one notable case, a patient experiencing intense emotional distress due to the cessation of PD-1 therapy was calmed through a combination of compassionate dialogue and detailed explanations by both nursing and medical staff. In addition, nurses facilitated virtual communication between patients and their families to reinforce a sense of connectedness and support, ultimately improving patient morale and cooperation.

### 3.5 Discharge planning and continuous care

Upon stabilization and discharge, patients were provided with detailed instructions for the gradual tapering of corticosteroids to avoid rebound phenomena. The nursing team prepared individualized medication schedules and provided written documentation outlining the dosage and timing of each medication. Patients and their families were educated on the importance of adhering to these schedules and advised to seek immediate medical attention if symptoms such as fever or chest tightness occurred, as these could signal steroid-related complications, including pneumonia.

**Figure 2:**
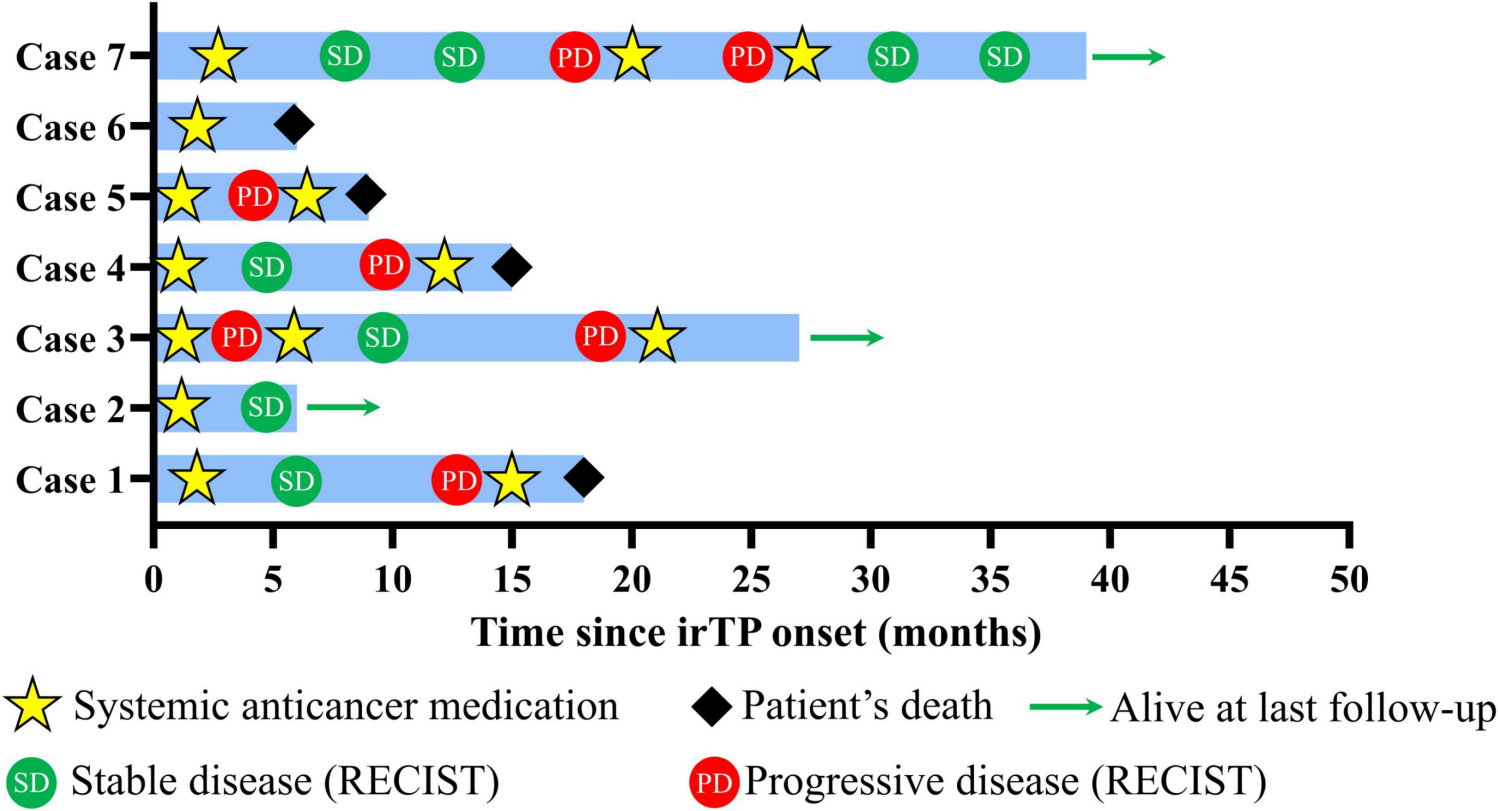
Swimming plot showing patients’ disease course. Tumor response was evaluated according to RECIST criteria. Briefly, the sum of longest diameters (SLD) of the target lesions in the follow up scan by computed tomography (CT) was calculated. PD indicated over 20% increase of SLD compared to smallest SLD in the study, while SD indicated less than 30% decrease of SLD or less than 20% increase of SLD.

Patients were also instructed to continue monitoring their vital signs and laboratory values, such as complete blood counts, electrolytes, and liver and kidney function tests, with regular follow-ups arranged at nearby hospitals. Detailed instructions were given regarding lifestyle modifications aimed at reducing bleeding risks and promoting overall health, including guidance on avoiding trauma, wearing loose clothing, and adhering to a non-irritating diet. Moreover, patients were introduced to an “Internet Plus Nursing Service Platform” designed to offer continuous post-discharge support from specialized orthopedic nurses and rehabilitation experts. This platform provided access to educational resources, scheduled virtual consultations, and a direct line of communication for any emerging concerns. The outcome of included 7 cases after continuous follow-up was illustrated in **Figure 2**.

## 4 Discussion

This case series highlights the occurrence of immune thrombocytopenia (TP) following PD-1 inhibitor therapy and underscores the dual challenges of managing immunotherapy-induced adverse events and implementing precision nursing care in clinical practice. The mechanism by which PD-1 inhibitors enhance antitumor immunity simultaneously predisposes patients to immune dysregulation (16). This dysregulation can lead to an unchecked activation of autoreactive T cells that target platelets (17–19). Furthermore, the concomitant use of bone-targeted therapies, such as denosumab, might further affect bone marrow function and contribute to a more severe or refractory course of thrombocytopenia.

Our findings reveal a clear temporal association between the initiation of PD-1 inhibitor therapy and the onset of thrombocytopenia. Notably, when compared with previous studies involving patients without bone metastasis (10–13), our cohort appears to experience a higher incidence and more complex clinical course of irTP. In metastatic bone cancer patients, the dual impact of tumor-related bone marrow involvement and potential denosumab-induced marrow suppression likely contributes to an elevated risk of spontaneous bleeding and other complications. This unique interplay underscores the need for heightened vigilance in this population. However, we must clarify that the current study cannot establish causality between denosumab and irTP.

Although hematologic irAEs are relatively infrequent compared to other immune-related toxicities such as those affecting the skin or gastrointestinal system, their clinical impact can be profound (13). While first-line management with corticosteroids and other immunosuppressive agents has generally proven effective (18, 20), these pharmacologic strategies must be balanced against the imperative of maintaining effective oncologic treatment, especially in patients with advanced malignancies (21). In cases where thrombocytopenia is refractory or severe, alternative strategies—such as the use of thrombopoietin receptor agonists—may be warranted, with close monitoring by nursing staff to assess both efficacy and potential side effects.

The present study emphasizes that effective management of irAEs requires an interdisciplinary approach. Furthermore, a critical aspect of managing PD-1 inhibitor–induced TP is the role of precision nursing care (14). Nursing professionals are uniquely positioned to detect early clinical changes, given their frequent and direct interactions with patients. Through meticulous monitoring of bleeding signs, laboratory parameters, and overall patient well-being, nurses can facilitate early identification of thrombocytopenia and prompt initiation of appropriate interventions. Moreover, the nursing care provided extends beyond mere monitoring to include comprehensive patient education, which empowers patients to recognize early signs of adverse events and to seek timely medical attention (22).

The comprehensive approach described herein, which integrates both pharmacologic and nursing strategies, is instrumental in optimizing patient outcomes. The results of our study demonstrate that early recognition of PD-1 inhibitor–induced thrombocytopenia, coupled with prompt and precise interventions, can lead to favorable outcomes even in patients with advanced malignancies and bone metastasis. Despite these promising findings, our study has several limitations. The retrospective design and small sample size (n=7) limit the generalizability of our results and introduce potential selection bias. Additionally, the confounding effects of concurrent therapies, particularly the use of denosumab, may have influenced the severity of thrombocytopenia and are challenging to fully account for in this analysis.

Future studies should aim to elucidate the precise immunological mechanisms underlying PD-1 inhibitor–induced TP. Larger, prospective trials are needed to confirm our observations and to establish standardized protocols that integrate both pharmacologic and nursing interventions. Furthermore, the identification of predictive biomarkers could significantly enhance clinical decision-making by enabling the early identification of patients at high risk for irTP and guiding personalized treatment strategies.

## 5 Conclusion

PD-1 inhibitors offer significant therapeutic benefits for patients with advanced malignancies, including those with bone metastasis, yet they also carry the risk of inducing severe irAEs such as immune thrombocytopenia. Optimizing patient outcomes requires a comprehensive, interdisciplinary management strategy. Our study demonstrates that early detection, prompt pharmacologic intervention, and the implementation of precision nursing care are critical components in optimizing outcomes for patients experiencing PD-1 inhibitor–induced TP. For clinicians, we recommend a stepwise approach: Firstly, early recognition. Monitor for key signs such as unexplained bruising, petechiae, and rapid declines in platelet counts. Secondly, immediate intervention. Initiate high-dose corticosteroids at the first sign of thrombocytopenia, and escalate treatment based on patient response and severity. Thirdly, interdisciplinary collaboration. Establish an integrated care team—including oncologists, hematologists, immunologists, and nursing staff—to tailor treatment plans and ensure continuous patient monitoring. Finally, patient education. Empower patients through education on recognizing early symptoms and maintaining adherence to follow-up protocols.

Future research is warranted to clarify the immunological mechanisms underlying PD-1 inhibitor–induced thrombocytopenia, particularly the role of bone marrow suppression and the impact of bone-targeted therapies like denosumab. Prospective studies are needed to validate standardized, evidence-based protocols for managing irTP. Moreover, research into predictive biomarkers remains a key priority to identify patients at risk, thereby informing personalized treatment strategies and optimizing overall care.

## Data Availability

The raw data supporting the conclusions of this article will be made available by the authors, without undue reservation.
